# Cases of Fistula Perinei, with Remarks

**Published:** 1831-04-01

**Authors:** 


### VIII.

### ST. GEORGE'S HOSPITAL.

### Cases of Fistula Perinei.

The three last reports from this hospital have been devoted to the consideration of injuries of the head. Circumstances prevent us from continuing it at present, as we had intended, but we shall endeavour to resume it at the earliest opportunity. We may take the present opportunity of relating a few cases of fistula perinei that have lately been treated at the hospital. We have nothing new to offer on such a disease, indeed we wish that we had, for it constitutes too frequently an opprobrium to surgery and surgeons. The principles of treatment are obvious enough, the practice is too often unsuccessful. We may mention one or two slighter cases in the first instance.

Case 1.—*Fistula anterior to Scrotum, after the passage of a Calculus per Urethram.* William Marshall, æt. 15, a delicate-looking boy, admitted, March 17th, 1830, under the care of Mr. Hawkins.

Fistulous opening leading into the urethra, in the integuments anterior to the scrotum—urethra much contracted anterior to the fistula—natural phymosis—health pretty good. Four months ago had retention of urine, and says that a stone was discharged per urethram. Some time afterwards, an abscess formed anterior to the scrotum, and the present fistula was the result. The treatment consisted in the regular passage of a catheter; and, some pyrexia arising, the exhibition of salines. On the 8th May, the report states that a catheter had for some time been kept in the bladder, and the fistulous opening was smaller. The edges, being very hard and contracted, were touched on the day mentioned with the potassa fusa. Within the last few days, an abscess had been forming in the right side of the perineum, near the anus. The boy stated that, two years previously, an abscess had formed in the perineum, and discharged itself into the urethra. In a few days the abscess was opened, and a little fetid pus discharged. The catheter was now retained in the bladder. Whenever it became obstructed it was withdrawn, and not re-introduced for a day or two; the edges of the fistulous opening were touched twice a week with the nitrate of silver. Little progress was made, the health began to suffer, the boy became rather hectic, and on the 6th June he was discharged, for the purpose of obtaining a change of air. There was every reason to suspect him of the vice of masturbation.

CASE 2.—*Fistula Perinei from tying a String round the Penis—Operation.*

Alexander Wilday, ætat. 9, admitted April 7th, 1830, under Mr. Brodie.

Last Christmas, in order to amaze his school-fellows, he tied a ligature round the penis, just behind the glans. He had previously natural phymosis. Much inflammation ensued beyond the ligature, which appears to have passed through the skin by ulceration, before it was cut away by a surgeon. Swelling and abscess at the root of the scrotum ensued, and the present fistula was the consequence. The fistulous opening is of moderate size, and situated just anterior to the scrotum. All the urine is discharged through it. There is contraction of the urethra anterior to the fistula. Prepuce, which is phymosed, thickened and redundant. Health very good.

On the 10th, the prepuce was divided, and the cut edges of integument, on each side, united by ligatures. This operation succeeded well enough for the phymosis. On the 13th, an operation was performed for the relief of the fistula. Its edges were pared with a scalpel, and brought together transversely by three sutures, which were made to avoid the urethra. The edges were in good apposition, and were so maintained by sticking-plaster. For two or three days the urine passed through the natural channel, and it was thought that union of the edges of the fistula would take place. We should mention that a catheter was kept in the bladder, but it occasioned a good deal of irritation. On the 16th, the ligatures were cut away; there was tenderness and soreness of the parts around, and it was difficult to determine whether any urine passed through the wound or not. A few more days determined that the operation was ineffectual, and urine issued through the fistula. It was attempted to retain a catheter in the bladder, but this occasioned so much irritation in the bladder, that it was withdrawn. As generally happens, the urine escaped by the side of the instrument. On the 14th, a small silver catheter, about three inches in length, with the extremity rounded like a probe, and perforated in the centre, was introduced into the urethra, and retained there by tapes; at the same time, the edges of the fistula were very gently touched with the actual cautery. The opening, however, did not contract beyond a certain point, and this plan answered no better than the preceding. After this, a series of graduated probes were employed to dilate the urethra, leaving the fistula alone. The probe was passed down the urethra daily, and retained in it for about a quarter of an hour. In November the lad was made an out-patient, the same system being still pursued. We cannot say that, when we saw him last, we perceived any great amendment. The edges of the fistula were contracted, and puckered, and indurated, and the penis somewhat constricted at the part.

CASE 3.—*Fistula Perinei after Contusion—various Operations—Death.*

Jas. Young, æt. 39, admitted, April 28th, 1830, under Mr. Keate.

Constant dribbling of urine from a fistulous opening, about the size of a pea, in perineo, exactly at the posterior root of the scrotum. From that to within half an inch of the anus is the cicatrix of a former incision, in the direction of the urethra; parts around inflamed from the contact of urine. Never passes urine through the penis, but it is projected with much force, and in a very small stream, from the fistulous opening, when he attempts to make water. Slight discharge from the orificium urethræ on taking cold—in coition, the semen is discharged through the fistula. Health tolerably good, but he does not feel strong.

He is a bricklayer, and has been for the last few years in Egypt, in the service of Mehmet Ali. Fifteen months ago he fell astride the hand rail of a staircase, and injured the perineum, considerable swelling of which ensued. Leeches, &c. were employed, and a French surgeon at Cairo attempted to introduce a catheter but failed. Effusion of urine into the cellular membrane on the inside of the left thigh succeeded, scarifications were employed, several attacks of retention of urine followed, and a variety of incisions and operations appear to have been resorted to. He has had the present fistulous opening for nine months; has been incapable of passing any water through the penis for five.

An attempt was made early in May, and another on the 10th, to pass an instrument into the bladder. This was not accomplished, the instrument not being introduced more than half an inch beyond the fistulous opening. On the 14th, Mr. Keate made an ineffectual attempt to find the portion of the urethra leading from the fistula to the bladder. A catheter was passed as far as the fistula, there cut down on, and the channel of the urethra thus exposed. Beyond this, however, all was obscurity, for a false passage appeared to debouche from the left of the fistula to half an inch beyond it, whilst the remainder of the urethra presented a semi-cartilaginous condition. The wound was dressed with lint and the patient removed to bed. In the afternoon the house-surgeon succeeded in introducing a small gum catheter from the fistula into the bladder. No water was discharged at first, but in the course of half an hour about a pint and a half came away, and by 12 o'clock next day nearly two quarts. By some accident the catheter slipped out of the bladder, and could never afterwards be re-introduced. On the 17th one or two unsuccessful attempts were made for this purpose. At 5 p. m., he had a rigor, followed by vomiting, anxious countenance, and low fever. There were some swelling and hardness around the wound, the surface of which looked sloughy. The sickness persisted on the 18th, and he was ordered salines. On the 19th he only felt nausea. Two unsuccessful attempts were made to introduce a catheter. In the night he had a chill, and next day there was more fever, of a low type. He had much scalding and irritation in the passage of the urine through the fistula, and an abscess appeared to be forming in the perineum. The matter and urine escaped through the fistulous opening, and did not require an incision. The tongue was at this period slightly aphthous, red and glazed at the tip. In the morning of the 25th he had a rigor with nausea, and still more prostration. A dull erysipelatous redness, with much tenderness and some tumefaction had appeared about the perineum and commissure of the thighs. The prepuce was a good deal tumefied, which it had been for two or three days. No doubt the redness of the cutis was indicative of effusion of urine into the subcutaneous cellular membrane. He was ordered an oily injection, and red wine.

On the 26th scarifications were resorted to. The patient was put on bark and ammonia, red wine, and brandy. To check the sickness a mustard poultice was placed on the epigastrium, but it did not answer. Next day the symptoms were very low, and there was a disposition to hiccup. The discolouration of the integuments was somewhat diminished. An incision was made on the right of the wound in the perineum, but no fluid was discharged. An unsuccessful attempt was made to pass an instrument from the wound into the bladder. On the 28th, a fresh effusion into the scrotum—excessive irritability and restlessness—general symptoms of typhus—occasional vomiting of greenish fluid. More scarifications were made in the scrotum, the cellular membrane of which looked white and sloughy, and was infiltrated with urine. The scarifications were repeated in the evening. On the next day there was considerable tumefaction and pain on pressure above the pubes and towards the left groin. The patient was very low indeed, and the retching continued, with some pain at the umbilicus. At midnight he died.

*Sectio Cadaveris.* A little more serum than usual in the pelvis—no inflammation of the peritoneum. Liver pale and very flabby — kidneys very flabby and pale—pelvis of the ureters and infundibula enlarged, their lining membrane thickened and bearing marks of chronic inflammation — ureters dilated and their parietes thickened — both ureters and pelvis of kidneys containing a mixture of pus and urine.

Bladder contracted yet still capacious; much thickened; its inner membrane corrugated; containing a mixture of pus and urine. In the prostate a considerable collection of pus, but not in a distinct cyst or abscess being contained in the ducts and structure of the gland. Urethra, from the bladder to the fistula perinei, very capacious. At the latter part it became so contracted that no instrument could be got from the fistula into the bladder.

No effusion of urine in the deep cellular membrane of the perineum or pelvis — some effusion, and a softened state of the tissue in the subcutaneous cellular membrane over the pubes and right groin.

Thoracic viscera healthy. Cranium not examined.

Case 4. *Urinous Abscess ending in Fistula Perinei, after Stricture for 18 years—Death.*

Robert White, æt. 59, admitted July 14, 1830, under the care of Mr. Babington.

Swelling, the size of a duck's egg, in the centre of the inferior part of the scrotum, and having the characters of abscess—no swelling of the perineum. General symptoms rather low and typhoid—looks unhealthy. Has had stricture for 18 years, and bougies have been frequently introduced. The present symptoms have been coming on for a fortnight or three weeks.

The swelling was punctured with a lancet, and foul urinous matter was discharged. There was much lymph deposited at the sides of the abscess. A small catheter, without a stilet, was passed with little difficulty into the bladder. It appeared to pass through a gristly false passage, and some pus escaped through it previous to the exit of the urine. The quantity of the latter was remarkably small. The catheter was kept in the bladder at night, and a poultice was applied. Low symptoms set in, and the patient was ordered the diffusible stimuli, with wine and porter. The abscess in the scrotum, which was extensive, was syringed out with the solut. sod. chlor. and stuffed with carrot poultice. The instrument was passed into the bladder without difficulty night and morning, but the urine was always remarkably scanty and at times contained pus. The symptoms became lower and more typhoid, there was a tendency to nausea and to diarrhœa. Before his death, which occurred on the 23d, the instrument, introduced into the urethra, passed out at the wound in the scrotum.

*Sectio Cadaveris.* Body not excessively emaciated.

Kidneys large, pale, flabby, mottled in colour, with small yellowish spots in their structure. On section they looked not unlike Castile soap, but were paler. Some portions were injected with blood-vessels; others exhibited a deposition, apparently of partly organized lymph; the distinction of cortical and medullary substance was obscured. The whole exhibited, no doubt, a specimen of chronic inflammation of the kidneys. The mucous membrane of the pelvis and ureters was thickened, injected, with some lymph upon its surface. The ureters were not dilated. Bladder small, contracted, thickened, its inner membrane in a state of chronic inflammation. Urethra much dilated to the bulb. Beyond this the proper canal of the urethra was entirely obstructed, but a false passage extended from anterior to the obstruction, along the side of the natural channel, and opened into the bladder beneath the caput gallinaginis. The obstructed part of the urethra was of a gristly hardness. The tunica vaginalis of the testis was united with the thick walls of the sloughy scrotal abscess, which, of course, communicated with the urethra. The testicles were healthy.

There was nothing particular in the thorax, and the cranium was not examined.

Case 5. *Numerous Fistulous openings in the Perineum—Stricture for 20 years—Death.*

William Lord, æt. 68, admitted June 2d, 1830, under the care of Mr. Babington.

Has numerous fistulous orifices in the perineum, scrotum, (especially the right side,) and one over the right abdominal ring, through all of which the urine escapes. Aspect squalid and cachectic. By his own account, no urine has passed through the meatus for nearly ten years, during which time he has had the fistulæ. Has had stricture for twenty years.

On the 5th, the sinuses in the perineum were laid open, in order to give a more ready exit to the urine, and prevent it from passing along the sinuses upwards to the pubes. On the 11th, he had two very severe rigors; the integuments of the perineum were red, and the wounds were looking sloughy. He was ordered æther and camphor, with poultices. On the 14th, the cellular membrane of the perineum and posterior part of the scrotum was evidently infiltrated with urine, and the former was inclined to slough. He had been sick in the morning. Yeast poultice—bark and liq. op. sedativ. On the 15th he was extremely low. The cellular membrane of the perineum was sloughing, and the urinous infiltration was extending in the dependent parts of the skin, with an erythematous blush of the integuments. Wine and brandy, a muriatic acid lotion, and a poultice were ordered. The sloughing in the perineum was arrested, and the general symptoms were for a few days mitigated. In the afternoon of the 17th he had a rigor, and from this till the 20th he gradually declined. The symptoms were low and typhoid, he had a feeble intermitting pulse, a dry glazed tongue, and much despondency. The fistulous sores in the perineum were sloughy, with red inflamed cutis around them. In the evening of the 20th he died.

*Sectio Cadaveris.* Body emaciated. Integuments of whole perineum removed by ulceration and sloughing. The latter had not penetrated beyond the subcutaneous cellular membrane, but sinuses ran more deeply in various directions. One wound round the penis on the left, and debouched into a large opening on the right side of the pubes. Another passed between the left crus penis and corpus spongiosum. The anterior portion of the urethra ended about the root of the penis in a perfect cul-de-sac. Beyond this, for an inch and a quarter, was a solid callous mass, in which the corpora cavernosa and spongiosum were blended and no canal remained. About this spot were numerous sinuses.

The bladder was very capacious, not much thickened, free from ulceration. The membranous part of the urethra was dilated. About two inches anterior to the caput gallinaginis the membranous part of the urethra ended in two large apertures leading to the perineum near the arch of the pubes. These openings, originally formed by ulceration, led through the upper part of the urethra, for here, the crura having diverged, the corpus spongiosum was not surmounted by the corpora cavernosa.

Kidneys small, puckered. The left studded with small vesicles, and having one or two soft portions not unlike the medullary disease. The right kidney was in much the same state, but exhibited more of this altered structure, and in a few places there were something like the scrofulous tubercles in its structure.

Liver very soft and of nutmeg colour. Heart flabby, rather large.

We might mention more instances of fistula perinei, but that would be unnecessary. Enough has been said to prove that these cases are extremely troublesome, too often fatal. The principles of treatment, surgical treatment, are two; first, to restore the urethra as nearly as possible to its natural condition: secondly, to heal the fistula by means directed more particularly to it. All authors have agreed that a great many fistulæ in perineo may be cured by simple dilatation of the urethra. Suppose, for instance, that a man has a permanent stricture of no great severity. From some cause or other, retention of urine is induced, the urethra inflames and gives way behind the stricture, there is an urinous abscess in the perineum, it is opened, and there is a sinus or an abscess communicating with the urethra. There can be no question, that if such a case be treated early, mechanical dilatation of the urethra will alone effect a cure of the fistula, if such it can be called. We might mention several such cases, but they must be familiar to all who have seen much of hospital practice. If the fistula be of some standing, dilatation of the urethra is not so efficient, and it then becomes necessary to rouse the callous borders of the fistula itself, and stimulate them to contraction and cicatrization. In the first case upon our list the urethra was dilated tolerably freely, yet the fistula could not be got to close. What is to be done in such a case? The indications are obvious, the means of effecting it not always successful. Surgeons have laid open the fistula, cut it across, scarified the edges, pared them and brought them together, employed stimulating applications, caustic, the actual cautery, &c. Each has occasionally succeeded, all have occasionally failed. Much depends on the state of the constitution, and after the most assiduous surgical attentions have been foiled, invigoration of the health has brought a cure.

In the third grade of fistula there may still be no stricture, or at least not a very severe one, but much loss of substance of the urethra exists. To such, the principle of the Taliacotian operation appears to be peculiarly applicable, and in such this plan proved sncccessful in the hands of Sir Astley Cooper and Mr. Earle.

In the fourth variety, the fistula or fistulæ are more or less ancient, the stricture impermeable, or nearly so. Here it is obvious that the urethra must receive our first and chief attention, and here it is that surgeons are constantly baffled in their operations. The channel of the urethra is nearly or entirely obliterated anterior to the stricture, and no bougie nor other instrument can be passed into the bladder. Whilst the urethra continues thus obstructed, the fistula cannot heal, and should not heal; if the obstruction is removed, it may. But how to remove it? We are not compósing a surgical dictionary, and need not enumerate the plans that have at various times been adopted. The operation commonly performed consists in the introduction of an instrument down to the stricture, cutting on that instrument, endeavouring to ascertain the orifice of the portion of the urethra which leads to the bladder, and attempting the passage of an instrument from that orifice into the bladder. If this be effected, time and patience will enable the surgeon to introduce a catheter from the penis into the bladder, to dilate the urethra, and restore its calibre. Then the wound heals, and perhaps the fistula also. Such was the operation attempted in the case of Young, and such we have witnessed on other occasions. It sometimes succeeds but more frequently fails, and the anatomy of the parts, with the changes produced by long-continued stricture, sufficiently explain the causes of failure.

A hospital surgeon, and we believe we may safely suspect that Mr. Mayo is he, has proposed a different method.

The operation, says this able surgeon, of dividing a stricture in the urethra, *situated in the usual seat of stricture*, viz. at the junction of the bulb with the membranous portion, may be performed with great quickness in the following manner:—

Introduce a middle-sized staff into the urethra till it reaches the stricture, then feeling the end of the instrument from the perineum, cut down upon it, the incision made through the integuments being one inch and a half in length, and parallel and close to the raphe, the incision into the urethra being half an inch in length. Withdraw the staff, after having exactly seen and felt where it points when held in the usual position towards the bladder; then thrust the scalpel forward in the line of the contracted and indurated urethra, one half or three-fourths of an inch. Next introduce along the urethra a middling-sized catheter, and try if it will pass into the bladder; it probably will; but if it still be obstructed, withdraw it a little, and lengthen the incision that has been already made, if the part beyond be firm and cartilaginous; or from the point where the previous incision began, thrust the knife half an inch forwards, with the edge directed upwards, instead of transversely, as before; the catheter will afterwards certainly find a ready passage onward to the bladder.

The urethra *beyond the stricture* is sure of being opened by incisions made in this manner, provided the operator possess a correct anatomical knowledge of the parts, as that portion of the canal is always capacious in such cases, having been dilated by the long-continued pressure of the urine. Those who fail in performing this operation, fail from want of boldness, losing time in endeavouring to find a channel through the stricture by means of a probe or small catheter after the urethra is cut into, which may serve as a guide in the division of the stricture. Such a guide is not needed by one who relies upon sufficient anatomical knowledge, nor should be thought of by the operator, if, as is likely, he has already found the urethra impervious to the smallest instruments.

The operation is concluded by fixing the silver catheter in the bladder. The silver catheter had better be removed in forty-eight hours, and an elastic one substituted, which is to be changed for another every day subsequently, till the wound has cicatrized; at which time a urethra is left, which has indeed some tendency to contract, but which requires only, for the prevention of this consequence, an occasional introduction of a bougie.

This proposal is highly deserving of attention, and we do not doubt that ere long opportunities will offer of testing its merits.

The fifth variety of perineal fistula is we fear beyond the reach of art. Examples of it will be found in the cases of White and Lord, the 4th and 5th upon our list. In this the stricture has existed long, and produced much alteration of the urethra, the fistulæ are perhaps numerous, the cellular membrane of the perineum disorganized, and, which confers its distinctive character on this variety, the bladder or kidneys, or both, are diseased. Under such complications what can be expected? A cure, alas, is beyond the reach of human means, and palliative measures are all that can be safely adopted.

The divisions which we have made of perineal fistulæ are arbitrary, and like all such divisions, objectionable in some instances. But we venture to believe that in a practical point of view they are neither unnecessary nor unjust. It would require far more experience than that to which we can lay claim, to treat even such a subject as this in a precise and yet comprehensive manner.

